# Bronchoscopic management of solitary bronchial myelolipoma: a case report

**DOI:** 10.1186/s12890-019-0910-y

**Published:** 2019-09-02

**Authors:** Hyun Sung Chung, Kyu Min Lee, Jung Seop Eom, Insu Kim, Seyeon Park, Jihyun Ahn, Ahrong Kim, Chang Hun Lee, Geewon Lee, Min Ki Lee

**Affiliations:** 1 Department of Intermal Medicine, Pusan National University School of Medicine, 179 Gudeok-ro, Seo-gu, Busan, 602-739 South Korea; 2 Department of Pathology, Pusan National University School of Medicine, Busan, South Korea; 3 Department of Radiology, Pusan National University School of Medicine, Busan, South Korea

**Keywords:** Myelolipoma, Bronchoscopy, Pulmonary atelectasis, Lung neoplasms

## Abstract

**Background:**

Myelolipoma is a rare benign tumor composed of mature adipose and hematopoietic tissues. Most myelolipomas are found in the adrenal glands, whereas intrathoracic myelolipoma is extremely rare. In particular, bronchial myelolipoma without the involvement of lung parenchyma has never been reported.

**Case presentation:**

A previously healthy 38-year-old male developed dyspnea and a productive cough. Computed tomography revealed an endobronchial mass at the right bronchus intermedius and subsequent atelectasis of the right middle and lower lobes. Flexible bronchoscopy found a total obstruction of the right bronchus intermedius due to an endobronchial tumor. Using a rigid bronchoscope, the endobronchial tumor was resected and the base of the tumor was additionally ablated with a diode laser to prevent recurrence. The removed endobronchial tumor was a 13 mm × 20 mm-sized oval-shaped mass and was pathologically diagnosed as bronchial myelolipoma. Chest radiographs, obtained on the day following the procedure, showed an improvement of atelectasis, and accompanying symptoms were immediately improved. Follow-up bronchoscopy performed after 12 months evidenced no recurrence of the bronchial myelolipoma.

**Conclusions:**

We used bronchoscopic intervention in patients with solitary bronchial myelolipoma and there was no evidence of recurrence.

## Background

Myelolipoma is a rare benign tumor pathologically composed of mature adipose and hematopoietic tissues [1]. It is usually a nonfunctioning tumor and is found mainly in the adrenal glands [2]. In general, myelolipoma is asymptomatic; however, it can grow and may cause hemorrhage or pain related to its mass effect [1, 3, 4]. Therefore, surgical resection is recommended to prevent possible complications of myelolipoma if the size of the tumor increases or the patient experiences symptoms [3].

Intrathoracic myelolipoma, including the mediastinum, is extremely rare [5]. In particular, all myelolipomas of the respiratory system have been found in lung parenchyma [5, 6] and diagnosed by autopsy or surgical resection [7, 8]. In addition, all cases in which surgical resection was performed underwent this procedure to resolve symptoms related to airway obstruction such as pneumonia, refractory coughing, or dyspnea.

Until now, solitary bronchial myelolipoma without the involvement of lung parenchyma has never been reported. Here, we report a very rare case of a solitary bronchial myelolipoma, which was treated by bronchoscopic intervention using rigid bronchoscopy and laser cauterization. In addition, we also describe its natural course after bronchoscopic treatment.

## Case report

**Fig. 1:**
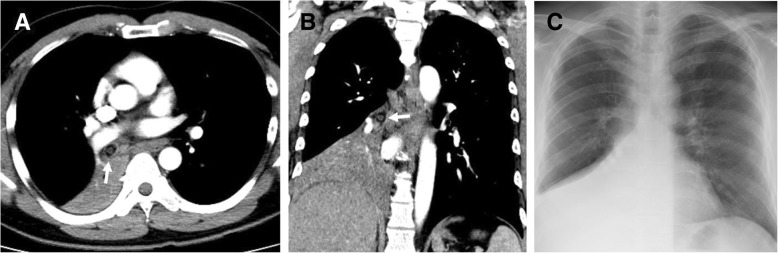
Initial radiologic findings of a previously healthy man with a solitary bronchial myelolipoma. (**a** and **b**) Initial axial and coronal chest computed tomography scan shows an endobronchial nodule of fat and a solid component in the right bronchus intermedius (arrow) combined with atelectasis of the right middle and lower lobes. (**c**) Initial chest radiograph shows atelectasis of the right lower lung zone

A previously healthy 38-year-old Asian male developed dyspnea and a productive cough. He was diagnosed at his local hospital with pneumonia and received antibiotics according to the treatment protocol for community-acquired pneumonia. In spite of an appropriate treatment regimen for pneumonia, his symptoms became aggravated. A computed tomography (CT) scan was then performed to evaluate the clear etiology of his symptoms and, consequently, an endobronchial mass at the right bronchus intermedius and subsequent atelectasis of the right middle and lower lobes were found (Fig. 1a and b).

**Fig. 2:**
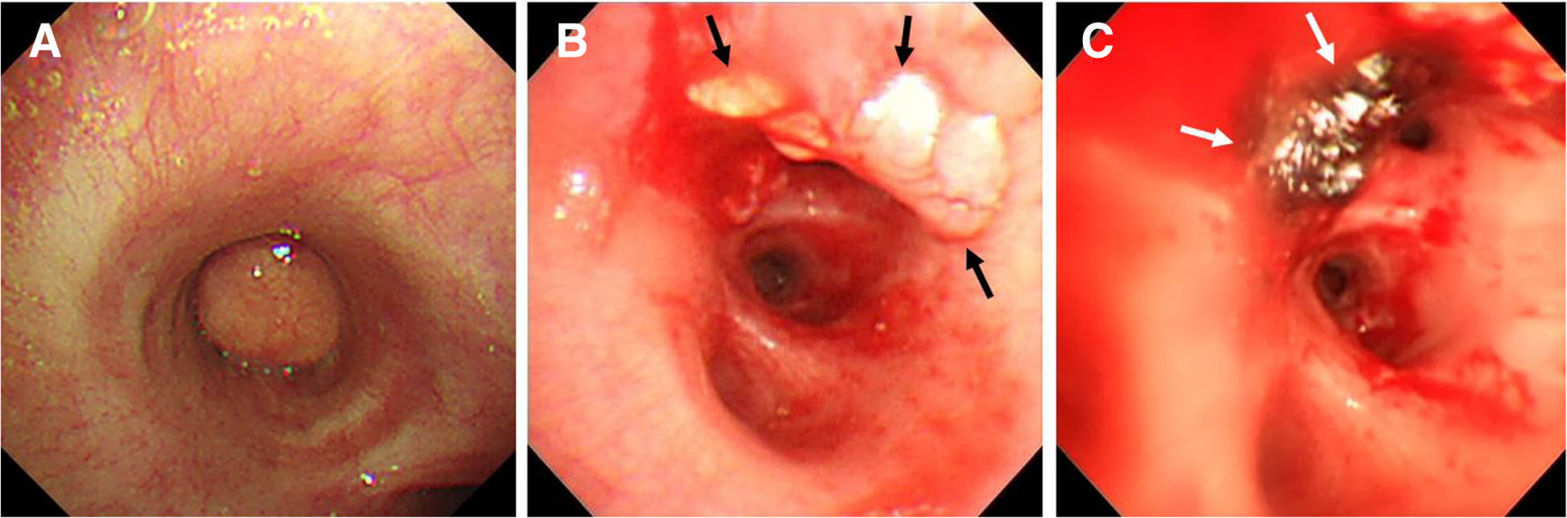
Bronchoscopic findings and treatment of a solitary bronchial myelolipoma. (**a**) Right bronchus intermedius is totally obstructed by a round endobronchial tumor. (**b**) Using rigid bronchoscopy, the endobronchial tumor was removed and the base of the tumor (black arrow) was identified at the anterior side of the distal right bronchus intermedius. (**c**) To prevent recurrence, the base of the tumor was ablated with a diode laser (white arrow)

**Fig. 3:**
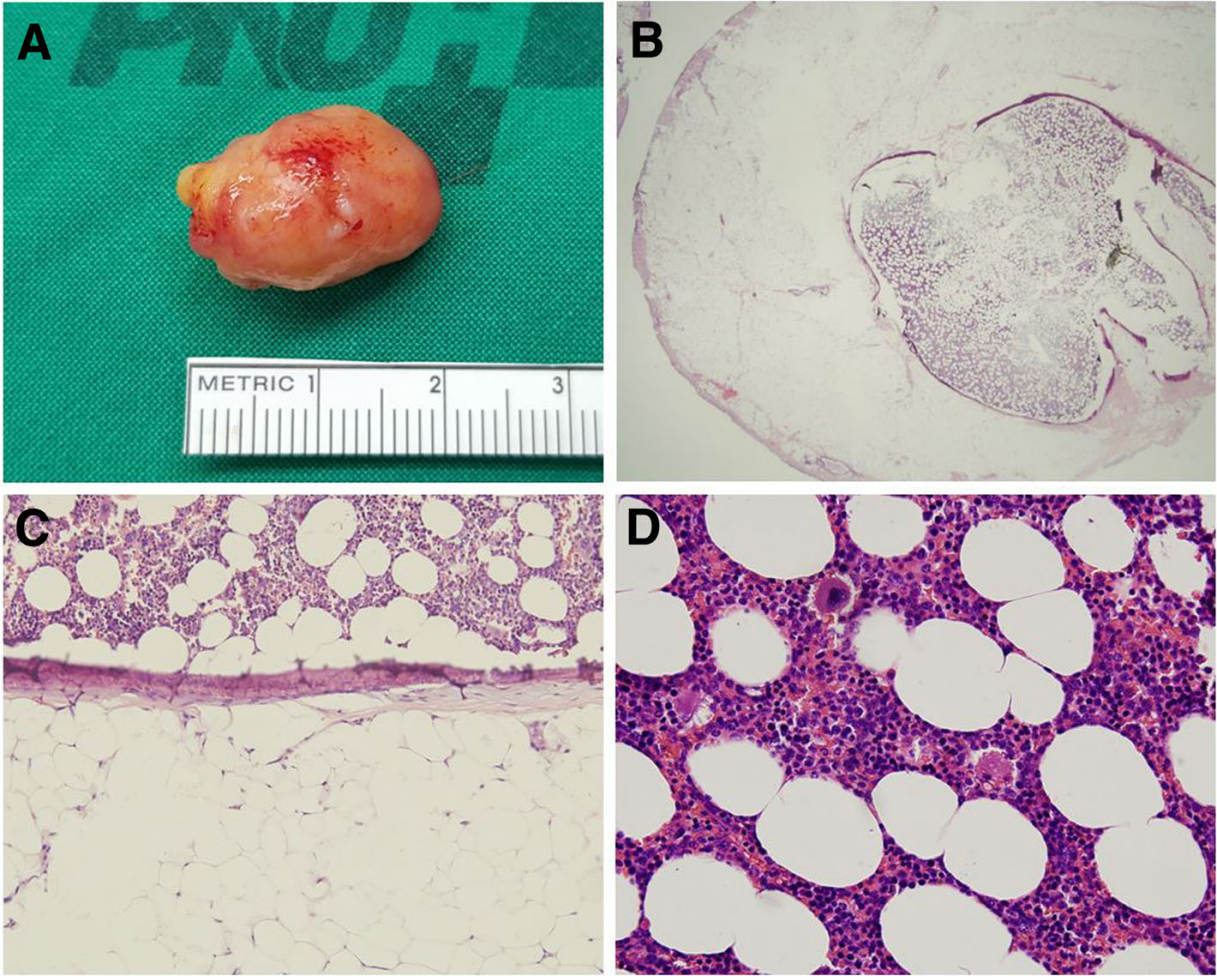
Macroscopic and microscopic findings of a solitary bronchial myelolipoma. (**a**) The endobronchial tumor of the right bronchus intermedius, removed using rigid bronchoscopy, was a 13 mm × 20 mm-sized oval-shaped mass. (**b**) A microscopic view of the solitary bronchial myelolipoma (hematoxylin & eosin [H&E] staining, × 25). (**c**) The tumor contained mature fat tissue with a central island of hematopoietic cells featuring an ossification rim (H&E staining × 100). (**d**) High-power field view shows normal trilineage hematopoiesis (H&E stain, × 400)

He was transferred to our hospital for further evaluation and treatment. His initial vital signs were stable and a chest X-ray showed right lower lung zone atelectasis (Fig. 1c). Treatment for obstructive pneumonia was continued using broad-spectrum antibiotics. Simultaneously, flexible bronchoscopy, which was performed to assess the endobronchial lesion, found that the right bronchus intermedius was completely obstructed due to an endobronchial tumor (Fig. 2a). To resolve the obstructive pneumonia and improve the patient’s symptoms, we scheduled immediate rigid bronchoscopy under general anesthesia to remove the endobronchial tumor. The tumor was resected using the sharp bevel of the rigid tube (Karl-Storz, Tuttlingen, Germany) (Fig. 2b). Thereafter, the base of the endobronchial tumor was ablated with a diode laser (Ceralas D50, Jena, Germany) to prevent recurrence (Fig. 2c). The removed endobronchial tumor was a 13 mm × 20 mm-sized oval-shaped mass (Fig. 3a). Pathologically, adipose tissues containing central hematopoietic tissues were identified and, consequently, bronchial myelolipoma was finally confirmed (Fig. 3). Radiologically, there was no parenchymal involvement of myelolipoma on CT. Clinically, the patient had no hematologic disorder such as anemia or thrombocytopenia, or any endocrine abnormalities.

**Fig. 4:**
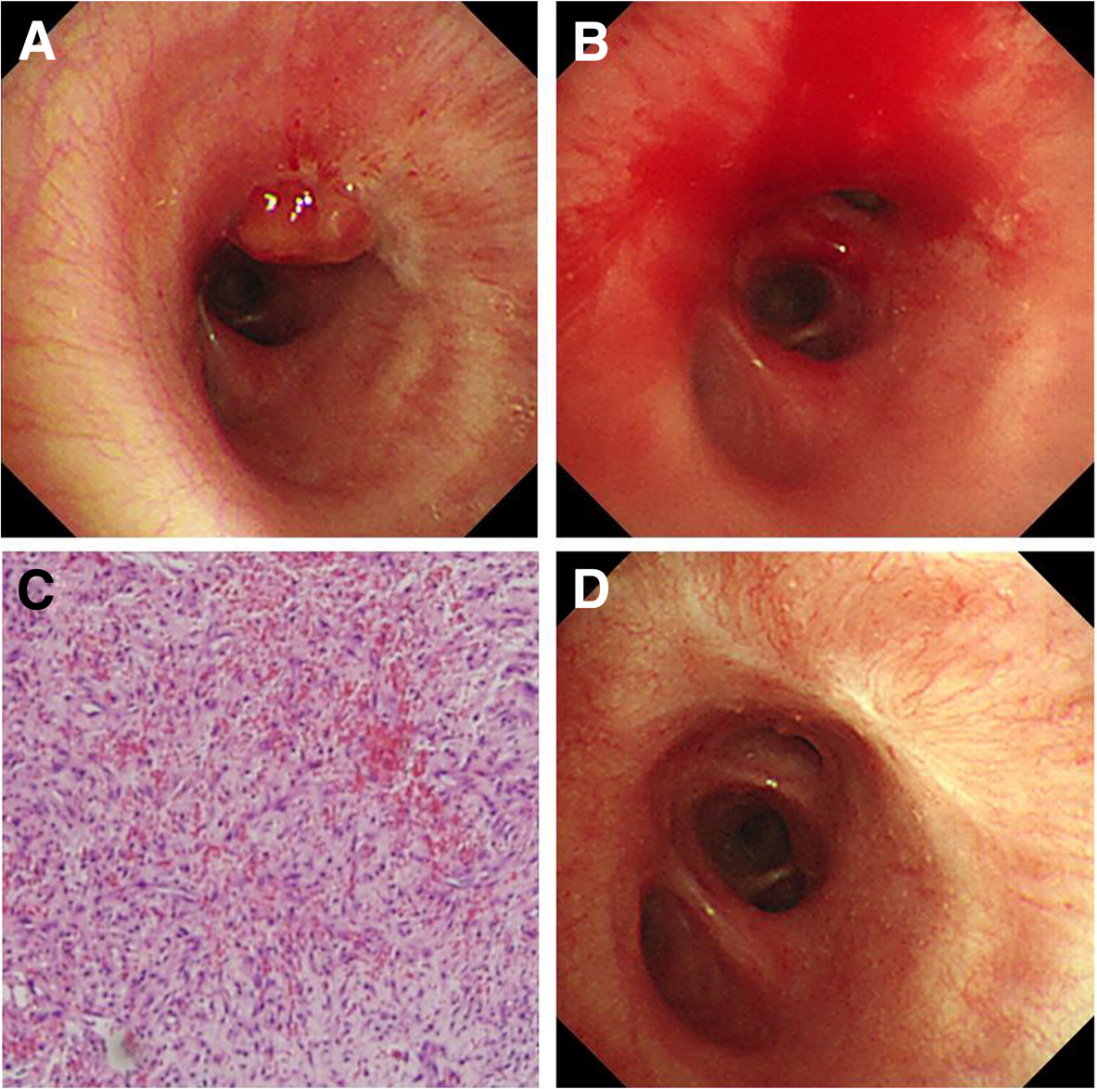
Follow-up bronchoscopic findings after removal of the solitary bronchial myelolipoma. (**a** and **b**) Granulation tissue overgrowth suspected at the previous base of the solitary bronchial myelolipoma was found on follow-up bronchoscopy 3 months later and was removed by biopsy forceps. (**c**) Microscopic findings of the granulation tissue show proliferation of fibroblasts and blood vessels with inflammatory cell infiltration (H&E stain, × 100). (**d**) Follow-up bronchoscopy performed 12 months after tumor resection revealed scar tissue on the previous tumor bed but no tumor recurrence

Chest radiographs, obtained on the day following the procedure, showed improvement of the right lower lung zone atelectasis, and accompanying symptoms such as cough, sputum, and dyspnea were immediately improved. After 3 months, a follow-up chest X-ray showed the disappearance of the right lower lung zone atelectasis. However, follow-up bronchoscopy showed granulation tissue overgrowth at the previous base of the solitary bronchial myelolipoma, and this was removed using biopsy forceps. Follow-up bronchoscopy performed 12 months later showed no recurrence of the solitary bronchial myelolipoma and no granulation tissue overgrowth (Fig. 4).

## Discussion and conclusions

Myelolipoma is mainly found in the adrenal glands and has a prevalence of 3–5% [1], whereas extra-adrenal myelolipoma has a prevalence of 0.08–2% and is mostly found in the presacral region or retroperitoneum [9]. Intrathoracic myelolipoma, which is predominantly found in the mediastinum, is rare and only ten intrapulmonary myelolipomas have been reported [6]. Until now, all reported intrathoracic myelolipomas were removed through thoracic surgery under general anesthesia [7]. To the best of our knowledge, this is the first report of a solitary bronchial myelolipoma that was removed through bronchoscopic intervention.

Endobronchial neoplasm can lead to bronchial obstruction and can cause symptoms such as cough, sputum, hemoptysis, or pneumonia [10]. Traditionally, bronchoscopy was used not only for directly observing the neoplasm in the bronchus but also for the removal of bronchial tumors in the large airway [11]. Previous studies have shown that bronchoscopy is useful as first-line management for the removal of benign lesions such as hamartoma, chondroma, leiomyoma, adenoma, and palilloma [11]. However, there is no previous report regarding the bronchoscopic removal of bronchial myelolipoma. In the present case report, the solitary bronchial myelolipoma was shown as a round, oval mass on flexible bronchoscopy, and resected smoothly using rigid bronchoscopy. In addition, there was no clinically significant bleeding during the bronchoscopic intervention.

Complete removal of an endobronchial mass via mechanical resection using a rigid bronchoscope alone is difficult. Clinically, it is impossible to decide whether there is a remnant tumor at the resection margin during bronchoscopic intervention. According to a previous study [12], additional laser ablation to the tumor bed was performed to prevent the recurrence of an endobronchial tumor.

McCracken et al. used a Nd:YAG (neodymium:yttrium-aluminum-garnet) laser to remove epithelial-myoepithelial carcinoma of the trachea [13]. Rai et al. used a diode laser for the complete removal of an endobronchial carcinoid tumor [14]. In our case, laser ablation was additionally performed to prevent recurrence after resection of the solitary bronchial myelolipoma. Although granulation tissue overgrowth was evident in follow-up bronchoscopy performed 3 months later, no recurrence of the bronchial myelolipoma was apparent. Twelve months after follow-up bronchoscopy, only scar tissue was evident on the previous tumor bed.

In conclusion, we found a solitary bronchial myelolipoma without involvement of the lung parenchyma, which was successfully treated with bronchoscopic intervention using rigid bronchoscopy and laser ablation.

## Data Availability

All the data supporting our findings is contained within the manuscript.

## Abbreviation

CT: Computed tomography
